# The Alzheimer's Disease Neuroimaging Initiative Clinical Core

**DOI:** 10.1002/alz.14167

**Published:** 2024-08-13

**Authors:** Paul S. Aisen, Michael C. Donohue, Rema Raman, Michael S. Rafii, Ronald C. Petersen, for the Alzheimer's Disease Neuroimaging Initiative

**Affiliations:** ^1^ Alzheimer's Therapeutic Research Institute University of Southern California San Diego California USA; ^2^ Department of Neurology Mayo Clinic Rochester Minnesota USA

**Keywords:** Alzheimer's disease clinical trials, amyloid, biomarkers, tau

## Abstract

The Alzheimer's Disease Neuroimaging Initiative (ADNI) Clinical Core is responsible for coordination of all clinical activities at the ADNI sites, including project management, regulatory oversight, and site management and monitoring, as well as the collection of all clinical data and management of all study data. The Clinical Core is also charged with determining the clinical classifications and criteria for enrollment in evolving AD trials and enabling the ongoing characterization of the cross‐sectional features and longitudinal trajectories of the ADNI cohorts with application of these findings to optimal clinical trial designs. More than 2400 individuals have been enrolled in the cohorts since the inception of ADNI, facilitating refinement of our understanding of the AD trajectory and allowing academic and industry investigators to model therapeutic trials across the disease spectrum from the presymptomatic stage through dementia.

**Highlights:**

- Since 2004, the Alzheimer's Disease Neuroimaging Initiative (ADNI) Clinical Core has overseen the enrollment of > 2400 participants with mild cognitive impairment, mild Alzheimer's disease (AD) dementia, and normal cognition.
- The longitudinal dataset has elucidated the full cognitive and clinical trajectory of AD from its presymptomatic stage through the onset of dementia.
- The ADNI data have supported the design of most major trials in the field.

## 1 INTRODUCTION

The Alzheimer's Disease Neuroimaging Initiative (ADNI) was initiated in 2004 to evaluate various clinical, imaging, and fluid biomarker measures to inform the field on the natural evolution of various measures during the development of Alzheimer's disease (AD) and therefore reflect the characteristics and longitudinal performance of individuals randomized to placebo arms in clinical trials in AD. The Clinical Core provides day‐to‐day coordination of all clinical activities at the ADNI sites, including project management, regulatory oversight, and site management and monitoring, as well as the collection of all clinical data and management of all study data. The Clinical Core is also charged with determining the clinical classifications and criteria for enrollment in evolving AD trials and enabling the ongoing characterization of the cross‐sectional features and longitudinal trajectories of the ADNI cohorts with application of these findings to optimal clinical trial designs.

Drs. Leon Thal and Ronald Petersen were the initial directors of the Clinical Core. In 2008, after the unexpected passing of Dr. Thal during the first phase of ADNI, Dr. Paul Aisen assumed the role of co‐director of the Clinical Core. Drs. Paul Aisen and Ronald Petersen have served as co‐directors of the Clinical Core since that time. The ADNI program consists of four distinct and overlapping phases.

## 2 ADNI1

The Clinical Core methodology for ADNI1 was developed in the setting of the then recently completed Alzheimer's Disease Cooperative Study (ADCS) trial on the evaluation of donepezil and vitamin E in mild cognitive impairment (MCI).^1^ This ADCS trial was the first clinical trial to venture into the clinical spectrum of predementia AD. MCI was characterized as a common predementia phase of the AD process and was being entertained as a new pharmacologic target for the AD spectrum. MCI is defined as persons who had a subtle degree of cognitive dysfunction, usually in the memory domain, but are otherwise functioning normally. They remain independent in their daily activities but are aware of an evolving cognitive deficit.

Previous work on MCI criteria had been laid out at the first Key Symposium in Stockholm in 2003.^2^, ^3^ These criteria were being adapted for various clinical applications, and ADNI chose to implement them for the recruitment of participants in clinical trials. MCI became the focal clinical group being recruited in ADNI1 and operationalized the following criteria: (1) memory impairment beyond what would be expected for aging and representing a change for that person, (2) relatively preserved performance in other cognitive domains, (3) preservation of activities of daily living, and (4) did not meet criteria for dementia.^3^ ADNI1 chose to use the amnestic MCI subset of individuals because this clinical phenotype most likely represented the earliest symptomatic manifestations of AD.^4^ However, it should be noted that the ADNI1 implementation of MCI criteria likely represented a more advanced stage of the condition than may be seen in observational research settings. The projected Clinical Core enrollment for ADNI1 included the following: normal cognition (*n* = 200), MCI (*n* = 400), mild dementia (*n* = 200). In the early iterations of ADNI1, the terms “AD” and “dementia” were used synonymously, but more recently, with the advent of biomarkers for AD, the distinction between AD and dementia has been clarified.^5^ Therefore, in this document, the term “dementia” will be used for the clinical, syndromic classification. Based on an ADCS MCI trial, the anticipated progression rate of participants with MCI to the dementia stage was estimated to be 16% per year.^1^ In fact, this conversion rate was observed in ADNI1 supporting the use of similar MCI criteria in interventional studies.^6^

The clinical criteria for ADNI1 cohorts considered cognitive symptoms, cognitive scores, and clinical assessments. With regard to a memory complaint, the cognitively unimpaired (CU) participants had none, while both the MCI and dementia participants reported cognitive concerns. Scores on the Mini–Mental State Examination (MMSE) for the three cohorts had overlapping ranges: both the CU and MCI participants had to score between 24 and 30, and the dementia participants 20 to 26, all inclusive. The Clinical Dementia Rating (CDR) global score was 0 for CU and was 0.5 for MCI with a mandatory score of ≥ 0.5 in the memory box, and the CDR global for mild dementia was 0.5 or 1. To document a memory deficit, delayed recall of one paragraph from the Logical Memory II subscale of the Wechsler Memory Scale‐Revised was used with cutoff scores based on education as follows (maximum = 25): CU ≥ 9 for 16 years of education, ≥ 5 for 8 to 15 years of education, and ≥ 3 for 0 to 7 years of education. For participants with MCI or dementia, the cutoffs were ≤ 8 for 16 years of education, ≤ 4 for 8 to 15 years of education, and ≤ 2 for 0 to 7 years of education. In addition, the participants were to be in good general health and approximately matched on age. The MCI participants had to be functionally intact and independent, thus not qualifying for the diagnosis of dementia. The dementia participants had to meet National Institute of Neurological and Communicative Disorders and Stroke—Alzheimer's Disease and Related Disorders Association criteria for “probable AD.”^7^

**RESEARCH IN CONTEXT**

1. **Systematic review**: The authors considered their own experience in the establishment and conduct of the Alzheimer's Disease Neuroimaging Initiative (ADNI) Clinical Core as well as relevant literature using PubMed.
2. **Interpretation**: We provide an overview of the rationale for Clinical Core activities and review the impact on the field of Alzheimer's disease therapeutic research.
3. **Future directions**: We allude to the plans under way for Clinical Core work on ADNI4.

## 3 ADNIGO

In 2010, ADNIGO was initiated to establish a new cohort of individuals with less severe memory impairment criteria than seen in ADNI1. In ADNIGO, 200 persons with what was designated early MCI (EMCI) were enrolled. These individuals exhibited a memory deficit on the Logical Memory II recall of ≈ 1.0 standard deviations (SDs) whereas the individuals recruited in the ADNI1 phase had a memory score cutoff criterion, education adjusted, of ≈ 1.5 SD (Late MCI [LMCI]). ADNIGO thus established the concept of a milder subset of MCI participants based on episodic memory performance and provided longitudinal data to inform consideration of such individuals for trials.

## 4 ADNI2

**FIGURE 1:**
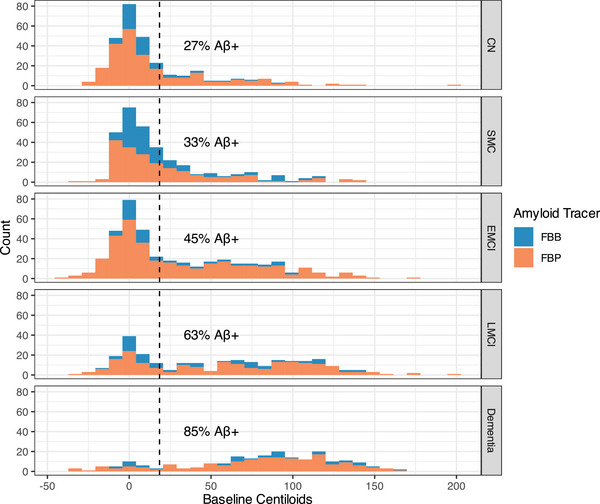
Amyloid PET results by clinical cohort in ADNI. Aβ+, amyloid beta elevation as indicated by PET scan; ADNI, Alzheimer's Disease Neuroimaging Initiative; FBB, florbetaben; FBP, florbetapir; PET, positron emission tomography.

**FIGURE 2:**
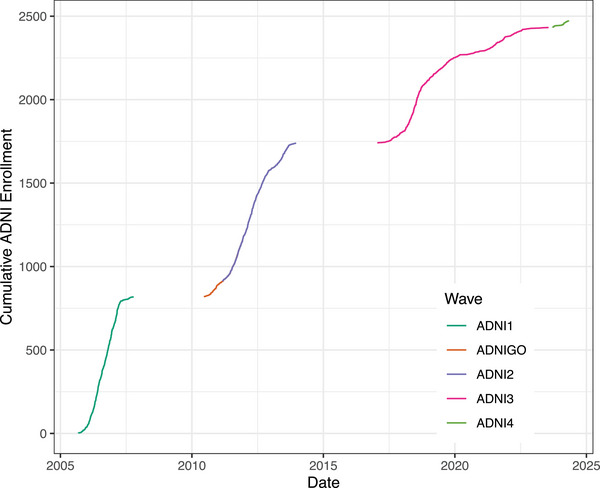
Transition rates between MCI cohorts and dementia and cognitively normal cohorts in ADNI. ADNI, Alzheimer's Disease Neuroimaging Initiative; MCI, mild cognitive impairment.

With the success of the enrollment of ≈ 200 persons with EMCI in ADNIGO, ADNI2 was designed to further characterize individuals along the clinical spectrum. With that intent, the cognitively normal subset of individuals was divided into those who were cognitively normal without a subjective complaint (*n* = 150) and a subset of individuals who were performing cognitively normally but had a subjective memory complaint (*n* = 100). In addition, another 100 participants with EMCI were recruited as well as a cohort of 150 LMCI participants and an additional 150 persons with mild dementia. This more granular characterization of the clinical spectrum proved to be useful in clarifying the relationships between baseline features and longitudinal progression. The biomarker profiles of individuals across the spectrum from normal to subjective memory complaints (SMC), EMCI, LMCI, and dementia were likewise revealing. In particular, amyloid positron emission tomography (PET) imaging results corroborated the clinical continuum (Figure 1). In addition, the rates of progression of the participants to the next severe clinical classification were approximately as expected^8^ (Figure 2); the biomarker profiles were consistent and neuropathological outcomes corroborated the clinical classifications.^9^

## 5 ADNI3

ADNI3 was initiated in 2016. However, as mentioned, the implementation of the more granular criteria was challenging, and consequently, the clinical criteria were collapsed for ADNI3 to three groups: CU, which included normal cognition and SMC; MCI, which included EMCI and LMCI; and mild dementia.

## 6 ADNI4

The Clinical Core in ADNI4 will maintain the same clinical criteria for enrollment—clinically unimpaired, MCI, and dementia—but will focus on understanding and evaluating the similarities and differences in the longitudinal characterization across a more inclusive sample given the increased participation from underrepresented populations. Working closely with the Engagement Core to recruit the underrepresented populations, the Clinical Core will continue to follow the cohorts longitudinally. The current clinical and demographic status of the ADNI population is shown in Table 1.

**TABLE 1:** Demographic summaries by ADNI wave.

	ADNI1 (*N* = 819)	ADNIGO (*N* = 131)	ADNI2 (*N* = 790)	ADNI3 (*N* = 692)	ADNI4 (*N* = 50)	Total (*N* = 2482)
**Baseline Diagnosis**						
CN	229 (28.0%)	0 (0.0%)	294 (37.2%)	374 (54.8%)	16 (37.2%)	913 (37.0%)
MCI	402 (49.1%)	131 (100.0%)	345 (43.7%)	237 (34.7%)	17 (39.5%)	1132 (45.9%)
Dementia	188 (23.0%)	0 (0.0%)	151 (19.1%)	72 (10.5%)	10 (23.3%)	421 (17.1%)
**Age**						
Mean (SD)	75.2 (6.8)	71.5 (7.9)	72.7 (7.2)	70.7 (7.4)	71.8 (7.7)	72.9 (7.4)
Range	54.4–90.9	55.5–88.3	55.0–91.4	50.4–90.6	55.5–85.2	50.4–91.4
**Sex**						
Female	342 (41.8%)	60 (45.8%)	379 (48.0%)	380 (54.9%)	34 (68.0%)	1195 (48.1%)
Male	477 (58.2%)	71 (54.2%)	411 (52.0%)	312 (45.1%)	16 (32.0%)	1287 (51.9%)
**Education**						
Mean (SD)	15.5 (3.0)	15.8 (2.7)	16.3 (2.6)	16.4 (2.3)	16.2 (2.5)	16.1 (2.7)
Range	4.0–20.0	10.0–20.0	8.0–20.0	10.0–20.0	12.0–20.0	4.0–20.0
**Ethnicity**						
Hispanic or Latino	19 (2.3%)	8 (6.2%)	31 (3.9%)	58 (8.4%)	7 (14.3%)	123 (5.0%)
Not Hispanic or Latino	794 (97.7%)	122 (93.8%)	755 (96.1%)	633 (91.6%)	42 (85.7%)	2346 (95.0%)
**Race**						
American Indian, Alaskan Native	1 (0.1%)	1 (0.8%)	1 (0.1%)	2 (0.3%)	0 (0.0%)	5 (0.2%)
Asian	14 (1.7%)	1 (0.8%)	14 (1.8%)	29 (4.2%)	7 (14.6%)	65 (2.6%)
Native Hawaii or Other Pacific Islander	0 (0.0%)	0 (0.0%)	2 (0.3%)	0 (0.0%)	0 (0.0%)	2 (0.1%)
Black or African American	39 (4.8%)	4 (3.1%)	34 (4.3%)	104 (15.2%)	11 (22.9%)	192 (7.8%)
White	762 (93.0%)	118 (91.5%)	728 (92.3%)	535 (78.3%)	27 (56.2%)	2170 (87.9%)
More than one race	3 (0.4%)	5 (3.9%)	10 (1.3%)	13 (1.9%)	3 (6.2%)	34 (1.4%)
**CDR‐SB**						
Mean (SD)	1.8 (1.8)	1.2 (0.7)	1.5 (1.9)	1.0 (1.6)	1.5 (1.8)	1.5 (1.8)
Range	0.0–9.0	0.5—4.0	0.0–10.0	0.0–10.0	0.0–7.0	0.0–10.0
**MMSE**						
Mean (SD)	26.7 (2.7)	28.3 (1.5)	27.4 (2.7)	27.9 (2.5)	27.1 (3.1)	27.4 (2.7)
Range	18.0–30.0	23.0–30.0	19.0–30.0	16.0–30.0	19.0–30.0	16.0–30.0

Abbreviations: ADNI, Alzheimer's Disease Neuroimaging Initiative; CDR‐SB, sum of boxes of the Clinical Dementia Rating; CN, cognitively normal; MCI, mild cognitive impairment; MMSE, Mini‐Mental State Examination; SD, standard deviation.

## 7 SUMMARY OF ADNI ENROLLMENT

**FIGURE 3:**
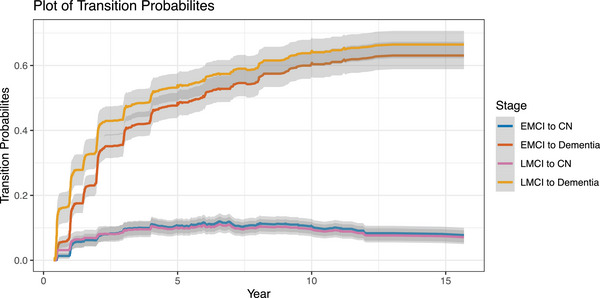
Cumulative enrollment in ADNI across all study phases. ADNI, Alzheimer's Disease Neuroimaging Initiative; CN, cognitively normal; EMCI, early mild cognitive impairment; LMCI, late mild cognitive impairment.

ADNI has enrolled > 2400 participants since its inception (Table 1). The demographics and clinical features have been similar to those in AD therapeutic trials. The individual enrollment figures are as follows: ADNI1, *N *= 819; ADNIGO, *N* = 131; ADNI2, *N* = 790; ADNI3, *N* = 692; and ADNI4, just beginning. The mean age has been 73.3 years, sex F/M (%) 48/52. The sum of boxes of the CDR (CDR‐SB) has adequately reflected the relative severity of the participants: CU, 0.0; SCD, 0.1; EMCI, 1.3, LMCI, 1.6; and dementia, 4.4. Figure 3 demonstrates the cumulative enrollment over the 20 years and shows recruitment by ADNI phase. The overall discontinuation rate in ADNI has been quite acceptable (5%–10% annually) and Figure 2 shows the progression rates among the various clinical classifications. All considered, the clinical cohorts have performed as expected.

## 8 ADNI AND AD CLINICAL RESEARCH

As discussed in the introductory paper for this issue and regular review articles,^10^, ^11^, ^12^, ^13^, ^14^, ^15^, ^16^, ^17^, ^18^ thousands of papers have been published on the ADNI cohorts and the clinical characteristics of the participants over time. The clinical construct of MCI has been included in the recent successful disease‐modifying therapies for AD with aducanumab,^19^ lecanemab,^20^ and donanemab,^21^ and MCI has been the most common clinical classification in these trials. The central importance of ADNI's clinical classification of participants to pivotal AD trials is clear.

In part due to the plethora of papers published on ADNI data, a recent evidence‐based medicine review of the literature on MCI performed by the American Academy of Neurology yielded > 11,500 studies on MCI. Nineteen Class I studies were documented demonstrating the prevalence of MCI to be between 10% and 20% in persons ≥ 70 years.^9^ The annual progression rate from MCI to dementia ranged between 5% and 20% per year with the most common figure being 10% to 15% per year. At that time, no pharmacologic therapies had been documented for MCI, but as mentioned above, that situation has evolved regarding MCI due to underlying AD, now a standard population in therapeutic trials.

The overarching purpose of the ADNI program continues to be to generate and share methods, standards, and longitudinal data to appropriately enable clinical trial designs that will bring effective therapies into clinical use. In 2023, the 19th year of ADNI, the first full US Food and Drug Administration approval of a disease‐slowing medication for AD was a major milestone of success for ADNI and the field of AD therapeutic research. In this 20th anniversary review, we consider contributions to this achievement.

### 8.1 Impact on the field

In the early years of this century, frustration was building over the failure of dozens of trials to move the field from the modest success of symptomatic treatments to the development of effective disease‐slowing interventions. Dozens of trials following plausible leads from preclinical and epidemiological research failed to find evidence of clinical benefit. Investigators hypothesized that the dementia phase of AD, marked by substantial irreversible neurodegeneration, might be too late for meaningful disease‐slowing therapy.^22^, ^23^ Shifting efforts to the predementia phase, MCI, presumably would enhance chances of success.

Initial trials in the MCI population were also frustratingly negative. Prior to the adoption of the research criteria using biomarkers to link MCI to AD,^24^ the MCI diagnosis was considered too heterogeneous to be appropriate for drug development; MCI trial designs were constrained to use time‐to‐dementia diagnosis as the primary outcome. Time‐to‐event designs require sacrifice of power compared to trials with continuous (e.g., cognitive performance or functional assessments) or pseudo‐continuous (clinical staging, as with CDR‐SB) primary outcomes.^25^

The general frustration with the lack of progress in AD therapeutics provided the key impetus to the design of ADNI. To increase the likelihood of successful trials, greater standardization of trial methods, from cognitive assessment to image acquisition and analysis, and clearer understanding of clinical and biological trajectories in AD were needed. The population to focus on would be MCI, presumably better than dementia for disease‐modification efforts. Elucidation of demographic and genetic influences on trajectories would enable efficient analysis methods.

As noted above, the basic design of ADNI involved testing standard criteria for MCI using the collection of demographic, clinical and genetic data, along with cognitive and functional assessment and biofluid (primarily cerebrospinal fluid) and imaging (magnetic resonance imaging and fluorodeoxyglucose PET) data. Two comparator groups, healthy age‐matched controls and individuals with mild dementia, were included. Long‐term follow‐up would provide data on the natural clinical and biomarker course of AD‐related MCI and enable efficient trial designs.

Accurate *ante mortem* diagnosis was not feasible in 2004; observational studies and clinical trials relied on clinical diagnosis of “probable AD” with substantial unavoidable errors. The diagnostic criteria for mild AD and MCI included clinical symptoms as indicated by global CDR of 0.5 (or, for mild dementia, 1) along with episodic memory impairment defined by a score on delayed paragraph recall testing at least 1.5 SD below an education‐adjusted norm. The cognitively normal control group required normal episodic memory and clinical assessment, and the absence of subjective cognitive concerns. There were no biomarker criteria for enrollment into any of the groups.

But in ADNI's first year, accurate molecular PET imaging of fibrillar amyloid deposits using C11 Pittsburgh compound B (PiB) arrived on the scene with appropriate fanfare.^26^ The ADNI team rapidly obtained additional funding to incorporate this valuable new tool into the schedule of assessments, not just for the MCI and mild dementia cohorts, but the cognitively normal group as well. The value of the PiB PET data cannot be overstated. Approximately 25% of the mild dementia cohort and 45% of the MCI group were amyloid negative, demonstrating the inaccuracy of clinical assessment even at the expert ADNI sites. The PET data enabled comparison of amyloid‐confirmed individuals with MCI and mild dementia to clinically similar amyloid‐negative individuals. The more recent addition of tau PET imaging enables longitudinal monitoring of both principal proteinopathies of AD further supporting the latest trial designs.^21^

A striking observation after the incorporation of PiB PET imaging into ADNI was the finding that more than one third of the normal cohort was amyloid positive, confirming earlier studies on the prevalence of brain amyloidosis. Anchoring clinical diagnosis of MCI to amyloid accumulation facilitated a new approach to early intervention trials.^27^ The ADNI dataset allowed exploration of the impact of amyloid accumulation in normal older individuals. Findings in this group of a relationship among amyloid, hippocampal volume, and cognitive trajectories lent strength to the idea that amyloid accumulation prior to symptom onset indicated an early stage of AD.^28^, ^29^, ^30^ This in turn led to discussions with regulators, general acceptance of presymptomatic stages of AD, and the design of trials of anti‐amyloid drugs in asymptomatic individuals.^22^, ^23^, ^31^

ADNI standardization of imaging methods and cerebrospinal fluid analysis, and near real‐time sharing of all ADNI data, provided powerful tools to drug development programs. The PRIME Phase 1b study, investigating the amyloid‐removing antibody aducanumab in a biomarker‐confirmed population of MCI and mild dementia was a major breakthrough in the field.^32^ Though designed to focus on tolerability and safety in a range of doses, clinical assessments (the CDR‐SB) and amyloid PET scans were included. The remarkable dose‐related reduction in amyloid accompanied by slowed worsening on the CDR‐SB gave credence to anti‐amyloid approaches when doses can be selected based on normalization of PET scans. Trials could be conducted with visibility into pharmacodynamic effects. While the aducanumab program was ultimately derailed by missteps in Phase 3, the lessons from PRIME provided a roadmap for the successful development of lecanemab and donanemab, both proven to slow clinical progression in early AD. Further, longitudinal ADNI data enabled the launch of presymptomatic trials of both agents, with hopes for larger beneficial effects. Indeed, it is likely that ADNI data fuel all modern drug development programs in the field.

The design of ADNI4 tries to address the major challenges and opportunities in AD clinical trials. First and foremost, AD trials have historically failed at inclusive enrollment. Building on the success of initial efforts toward the end of ADNI3, ADNI4 is committed to ensure effective outreach to and enrollment from underrepresented groups. In addition, the enormous advance in accurate plasma biomarkers of AD pathology enabled remote (i.e., community‐based rather than clinical site‐based) screening and phenotyping of potential participants in ADNI and AD. Thus, in ADNI4, tens of thousands of individuals will be recruited using web‐based capture of demographic and cognitive data and plasma phenotyping, with candidates selected to support the ADNI aims (the longitudinal study of inclusive populations of cognitively impaired and cognitively normal individuals). This will facilitate the latest trial designs in preclinical and prodromal AD.

## CONFLICT OF INTEREST STATEMENT

Dr. Aisen has research grants from NIH, Lilly, and Eisai, and consults with Merck, Roche, BMS, Genentech, Abbvie, Biogen, ImmunoBrain Checkpoint, Arrowhead, AltPep, and Neurimmune. Dr. Donohue has research grants from Eisai and Lilly, consults with Roche, and owns stock in Janssen. His wife is employed by Janssen. Dr. Raman has research grants from Eisai, Lilly, the American Heart Association, and the Alzheimer's Association. Dr. Rafii has research grants from Eisai and Lilly, consults with AC Immune and Ionis, and serves on an advisory board for Alzheon, Aptah Bio, Biohaven, Keystone Bio, Prescient Imaging, Positrigo, and Embic. Dr. Petersen has consulted for Roche Inc., Eli Lilly and Co., Eisai Inc., Nestle Inc., and Genentech Inc. Author disclosures are available in the supporting information.

## Supporting information

Supporting Information
